# Applications and Assessment of Social Media in Pediatric Orthopedics: Scoping Review

**DOI:** 10.2196/55360

**Published:** 2025-08-14

**Authors:** Yun Chen, Hui Ouyang, Gan Lin, Yating Li, Yichao Peng, Hongyu Zhong, Zijun Gao, Jinghui Yao

**Affiliations:** 1 Department of Outpatient Third Affiliated Hospital of Southern Medical University Guangzhou China; 2 School of Nursing Southern Medical University Guangzhou China; 3 Department of Pediatric Orthopedics Center for Orthopedic Surgery Third Affiliated Hospital of Southern Medical University Guangzhou China

**Keywords:** social media, pediatric orthopedics, smart medicine, health education, information dissemination

## Abstract

**Background:**

With the continuous advancement of science and technology, the demand for health knowledge about pediatric orthopedics is also gradually growing. The traditional paper-based and multimedia health education models can no longer fully meet the needs of society. Fortunately, the emergence of social media has mitigated the problem of insufficient medical education resources. However, there is currently relatively little published evidence on the use of social media in pediatric orthopedics.

**Objective:**

This study aimed to examine the current applications of social media in pediatric orthopedics and to evaluate the quality and readability of related online health information. Its purpose is to provide relevant evidence to promote the understanding and development of the field.

**Methods:**

This review followed the methodological framework of Arksey and O’Malley and the Joanna Briggs Institute reviewer manual. First, a literature search was performed in the PubMed, Embase, CINAHL, Web of Science, and Cochrane databases. The search time range was from the establishment of the databases to September 21, 2023. We endeavored to include research articles related to social media and involving pediatric orthopedics in the review. The literature was reviewed at the title, abstract, and full-text levels.

**Results:**

We included 35 of 3400 (1.03%) studies retrieved. Most of the articles used social media to help with medical staff and patient education and training (23/35, 66%) and to disseminate information (21/35, 60%), followed by helping medical staff collect data (8/35, 23%). Medical institutions and staff also used social media to increase attention (6/35, 17%), enhance social support (5/35, 14%), facilitate the recruitment of research participants (3/35, 9%), support professional development (3/35, 9%) and implement health intervention (2/35, 6%). Five general quality of information (QOI) tools, 7 specific QOI tools, and 6 readability tools were used in 12 studies analyzed for quality and readability, with overall quality being fair and readability exceeding the recommended level. According to the research data, people are increasingly interested in pediatric orthopedics on social media platforms and eager to obtain and learn relevant knowledge.

**Conclusions:**

This scoping review found that social media has a growing body of literature on pediatric orthopedic conditions and is playing an increasingly important role in knowledge dissemination and education. A variety of tools are being used for assessing the QOI, but little attention has been paid to the readability of the information. The QOI was largely fair, with readability above the recommended level. Future research should further explore the role of social media in pediatric orthopedics and continue to optimize QOI and information readability.

## Introduction

### Background

Before the internet, health education for pediatric orthopedics was mainly conducted with paper-printed media, such as newspapers, magazines, and books [1]. Then, technological progress and economic development led to the development and popularity of electronic media, such as telephones, radio, and television, which then evolved to impart health education through multimedia. Multimedia health education offers patients and their families an engaging, visually appealing, and memorable learning method that allows them to gain specialized condition knowledge, making health education more comprehensive, systematic, and standardized [2]. The emergence of the internet gave birth to social media. This stage is an era of network communication that mainly uses network platforms and social application software as the main communication methods, and the main body of communication has shifted from the original specific institutions and groups to diversification and equality. Everyone can be both an audience and a disseminator of information using social media [3]. Medical personnel’s approach has changed from passive inquiry to active education. A cooperative relationship in which doctors and patients participate and cooperate has been formed, which enhances the affinity with patients, improves patient satisfaction, and helps the treatment and nursing work to proceed smoothly.

The application of social media in health care is a bottom-up and patient-driven phenomenon that is changing people’s demand for and access to health information [4]. Nowadays, pediatric parents belong to a mostly younger generation group, and more parents can use the internet to obtain health information. A survey of parents of children undergoing pediatric surgery showed that 38.3% of parents had checked their children’s surgical issues online and 26.5% of parents had done online research before their first visit [5]; another study found that from 72% to 96% of parents who are ordinary internet users take their children to pediatric urology outpatient clinics [6]. The emergence of social media has brought about new changes in health education, forming a new win-win situation for medical institutions, medical personnel, and the general public. The widespread dissemination of information also has certain flaws, and the information is widely considered potentially incorrect or unauthorized. Unconfirmed information is flooding the field of pediatric orthopedics. Although there are scientific and objective suggestions, some parents lack an understanding of professional knowledge and have difficulty distinguishing the quality of the information; the content that is spread mainly involves normal variations in growth, such as flat feet, splayed feet, W-shaped sitting posture, and toe walking. These variations are considered abnormal and require some form of treatment, but children can improve on their own without intervention [7]. Published through various media without citations, information that lacks a scientific basis for research results and treatment recommendations may reduce the public’s trust in online health information and may even cause some people to question the professionalism of experts. Therefore, multiple parties need to maintain the network information-sharing platform.

Previous scoping and systematic reviews have already been published about the different uses of social media for health research [8,9]. However, they did not systematically identify and summarize all available evidence on the use of social media in pediatric orthopedics. By identifying the key characteristics and factors related to the use of social media in pediatric orthopedics, we can also better understand its potential impact on medical practice and patient care. The results of this review will provide valuable information for health care policy makers, educators, and practitioners, helping them to use social media tools better to improve the accuracy and accessibility of medical information.

### Objectives and Research Questions

This study aimed to explore the current use of social media in pediatric orthopedics through a scoping review and to address the following research questions:

1. What are the basic characteristics of social media in pediatric orthopedics researched in the literature?
2. What are the uses of social media in pediatric orthopedics researched in the literature?
3. What is the research on the quality of pediatric orthopedics health science–related information in the literature?
4. What is the research on the readability of information about pediatric orthopedics in social media?

## Methods

### Overview

The internal protocol, which was developed to direct the process and is accessible from the relevant author upon request, served as the basis for this scoping review. This methodology was chosen to summarize what was known and identify gaps based on the guidelines of scoping reviews developed by Arksey and O’Malley [10] and advanced by Levac et al [11]. This review adheres to the PRISMA-ScR (Preferred Reporting Items for Systematic Reviews and Meta-Analyses Extension for Scoping Reviews) [12].

### Inclusion and Exclusion Criteria

This review was guided by the population, concept, and context framework suggested by the Joanna Briggs Institute [13], which is described in Textbox 1 [14].

**Study inclusion and exclusion criteria.**

1. Population: Pediatric patients, caregivers, and medical staff, with “pediatric” defined as individuals aged <18 years.
2. Concept: Social media was defined as digital platforms enabling content creation, sharing, interaction, and virtual community engagement. This included social networking sites (eg, Facebook and Instagram), microblogging (eg, X), video-sharing platforms (eg, YouTube and TikTok), and interactive websites featuring user-generated content and community-building functions. Articles were determined eligible for inclusion if they discussed the use of social media, including but not limited to the use of social media for data collection, dissemination of information, education and training, health interventions, increasing attention, privacy ethics, professional development, recruitment of study participants, and social support. These categorizations are based on the social media for implementing evidence (SMILE) framework [14].
3. Context: The context was in orthopedics, including all types of musculoskeletal conditions. The context was not limited to any geographical location or particular musculoskeletal conditions. Studies involving multiple settings or cross-country comparisons were included.
4. Types of sources: We only included literature published from the establishment of the database to September 21, 2023.
5. There were no limits on the type of research (randomized controlled trials, cohort studies, and cross-sectional studies), except for systematic or scoping reviews, books, book chapters, or conference abstracts. Articles written in languages other than English were excluded. Articles that did not address both social media and pediatric orthopedics, or that did not follow the theme of the article, were excluded. Articles with multiple submissions that were not academically ethical were also excluded.

### Search Strategy

An experienced information specialist (YC) developed the comprehensive search strategies. A search of the literature was performed in September 2023 using 5 electronic citation databases: PubMed, Embase (Elsevier), CINAHL (EBSCO), Web of Science Core Collection (Clarivate Analytics), and Cochrane. The searches were conducted for the period from the establishment of each database to September 21, 2023, using index terms, where appropriate, and free-text terms to capture the following defined search terms: (1) social media, including both general terms and specific platform names and terms (eg, Twitter, tweet, Facebook, TikTok, and YouTube); (2) pediatric, including children of all ages; and (3) musculoskeletal conditions, including general terms for some common conditions. The full search strategy is summarized in Multimedia Appendix 1.

### Study Selection

The screening process was conducted using the PRISMA-ScR checklist (Multimedia Appendix 2) [12,15]. At least 2 reviewers (HO and GL) screened the titles and abstracts for assessment against the inclusion criteria. Following a preliminary screening of 56 reports, the full texts of the chosen studies were obtained for review, and 2 independent reviewers (HO and GL) conducted a thorough evaluation against the inclusion criteria. If full texts were inaccessible, the authors were contacted at least twice. If there were any disagreements between the reviewers, a third reviewer (YC) was engaged to mediate and resolve the disagreements.

### Data Extraction

One reviewer (HO) extracted all data from the included articles using abstract data, when available, with Zotero (version 6.0). The extracted data included article characteristics (title, abstract, year of publication, journal, country of the first author, and article type), study design, study object, study objective, study conclusion, social media platform, and preidentified categories related to the content for social media use. Extracted data were exported from Zotero into Microsoft Excel for analysis using descriptive statistics (eg, totals and percentages).

### Analysis and Presentation of Results

Guided by the approach of Zhao et al [14], the use of the social media for implementing evidence (SMILE) conceptual framework aimed to understand how social media was used as a knowledge-transformation strategy to help policy makers, health care professionals, and patients make health care decisions. Meanwhile, we applied the SMILE framework to analyze the resulting types of social media applications, which include data collection, dissemination of information, education and training, health interventions, increased attention, privacy ethics, professional development, recruitment of study participants, health care decision-making, recruitment of study participants, and social support. The SMILE framework is analyzed as shown in Table 1. Step 6, stakeholder consultation, as recommended by Arksey and O’Malley [10], was not completed because of limited resources.

**Table 1:** Social media use types within the social media for implementing evidence (SMILE) framework.

SMILE framework and categories		Descriptions
**Developers**		
	Medical institutions	Serve as content creators and distributors of public health policy, research updates, and health data on social media
	Medical staff	Serve as bridges between doctors and patients while acting as creators and distributors of content
	Public	Engage in information sharing and feedback as consumers and cocreators of content
**Messages and delivery strategies**		
	Health interventions	Use social media for health promotion and behavior change interventions
	Dissemination of information	Disseminate health information, research findings, and evidence through social media platforms
	Data collection	Use social media data for content analysis and data mining
	Professional development	Health professionals use social media for academic communication
	Education and training	Improve the knowledge of health care professionals and patients
	Privacy ethics	Address privacy protection and ethical issues when using social media for health interventions and research
**Recipients**		
	Policy makers	Understand public health needs and research findings, and develop relevant policies
	Medical professionals	Receive the latest research findings and educational content to improve professional competence
	Patients and families	Participate in health decision-making and behavior change as primary recipients of information
**Triggers**		
	Increase attention	Use social media alerts, campaigns, and advertisements to raise awareness of specific health topics and enhance the visibility of medical institutions and medical professionals
	Recruitment of study participants	Recruit research participants through targeted advertisements and posts on social media
	Social support	Use social media to create support groups to provide emotional support for patients
**Context**		
	Virtual technical environment	Consider the characteristics of social media platforms and user behavior
	Organizational environment	Consider the policies and culture within health care institutions and research organizations
	System environment	Consider the broader social, political, economic, and cultural environment
**Outcomes**		
	Knowledge enhancement	Measure the level of knowledge gained by the public and professionals through social media
	Health behavior change	Assess the impact of social media interventions on health behaviors
	Policy and practice change	Assess the impact of social media messages on policy and medical practice

## Results

### Study Selection

**Figure 1:**
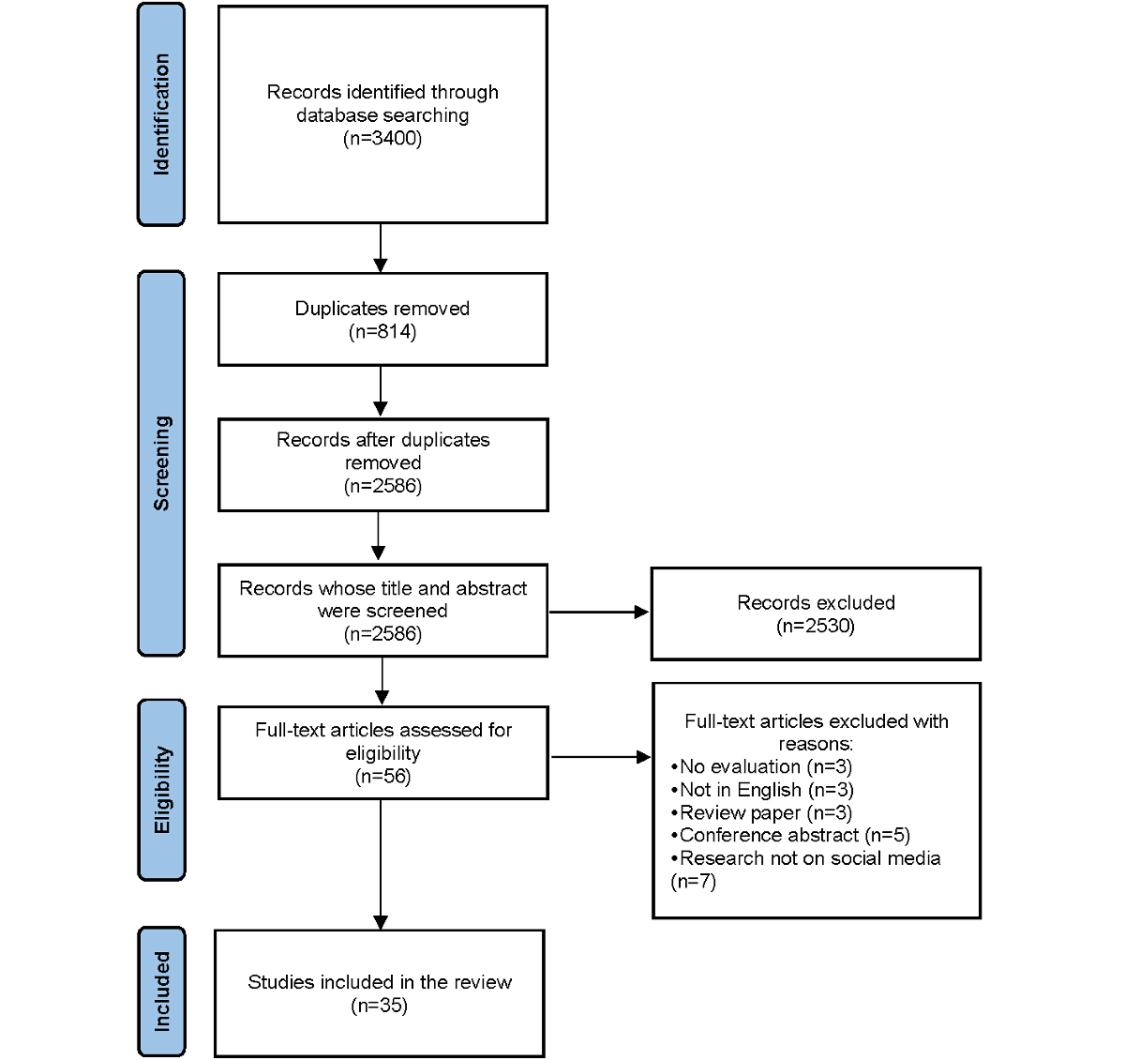
PRISMA for Scoping Reviews flow diagram of study search and selection.

On the basis of the initial search, 3400 articles were identified. After the removal of duplicates, 2586 (76.06%) articles remained for screening. During the title and abstract screening stage, 2530 (97.83%) articles were excluded. The remaining 56 (2.17%) articles were retrieved for full-text review. Of these, 21 (38%) articles were excluded for reasons detailed in Figure 1, resulting in a final inclusion of 35 (62%) studies. Data extraction was performed by 1 reviewer (HO, with a second reviewer (GL) assisting in the screening process.

### Characteristics of the Articles

The study identified 35 articles across 25 different journals. The number of articles published on pediatric orthopedics’ use of social media has increased significantly over time, ranging from 2013 to 2023. The number of publications continuously increased during the past 5 years, showing that social media use in pediatric orthopedics received growing attention from researchers. Influenced by the COVID-19 pandemic, the year 2021 was found to have the highest number of social media application studies on pediatric orthopedics; this number, however, started to decrease thereafter. Articles were most commonly published in the *Journal of Pediatric Orthopaedics* (7/35, 20%), *Spine Deformity* (3/35, 9%), *BMC Musculoskeletal Disorders* (2/35, 6%), and the *Journal of Pediatric Orthopaedics B* (2/35, 6%). The remaining journals published 1 paper each. An analysis of the articles’ research subjects found that researchers began to focus on a diverse group of research subjects, with almost half of the articles having social media–related subjects (18/35, 51%), which covered videos, websites, and tweets, followed by patients and their families (11/35, 31%), health care professionals (8/35, 14%), and finally, the general public (1/35, 3%). First authors of the included articles represented 9 different countries, most commonly the United States (13/35, 37%), China (5/35,14%), Canada (5/35, 14%), Ireland (3/35, 9%), Turkey (3/35, 9%), and Saudi Arabia (2/35, 6%). The remaining countries of origin are shown in Table 2.

**Table 2:** Characteristics of the included studies (N=35).

Characteristics		Studies, n (%)
**Publication year**		
	2013	2 (6)
	2014	0 (0)
	2015	2 (6)
	2016	1 (3)
	2017	2 (6)
	2018	3 (9)
	2019	4 (11)
	2020	2 (6)
	2021	10 (29)
	2022	3 (9)
	2023	6 (17)
**Country of first author**		
	United States	13 (3)
	United Kingdom	1 (3)
	Canada	5 (14)
	China	5 (14)
	Germany	1 (3)
	Ireland	3 (9)
	Israel	1 (3)
	New Zealand	1 (3)
	Saudi Arabia	2 (6)
	Turkey	3 (9)
**Design study**		
	Cross-sectional study	22 (63)
	Descriptive study	2 (6)
	Observational study	1 (3)
	Quasi-experimental study	4 (1)
	Randomized controlled trial	1 (3)
	Retrospective study	4 (11)
	Semistructured interviews	1 (3)
**Media platform**		
	Douyin	1 (3)
	Facebook	1 (3)
	Multiple	8 (23)
	Not stated	8 (23)
	Twitter	2 (6)
	Websites	5 (14)
	WeChat	3 (9)
	WhatsApp	2 (6)
	YouTube	5 (14)
**Condition**		
	AIS^a^	8 (23)
	Anterior cruciate ligament injury	1 (3)
	ARLs^b^	1 (3)
	Clubfoot	4 (11)
	DDH^c^	5 (14)
	Fractures	3 (9)
	Multiple	1 (3)
	Not specified	7 (20)
	Osteosarcoma	2 (6)
	Perthes disease	1 (3)
	Trauma	2 (6)

^a^AIS: adolescent idiopathic scoliosis.

^b^ARL: acetabular roof lesion.

^c^DDH: developmental dysplasia of the hip.

### Characteristics of Social Media Use in Pediatric Orthopedics

#### Type of Condition

Because of the topic’s need for inclusion of pediatric orthopedics, a total of 27 articles included in the screening mentioned terms for specific conditions and studies related to social media. These included adolescent idiopathic scoliosis (AIS; 8/27, 30%), developmental dysplasia of the hip (DDH; 5/27, 19%), clubfoot (4/27, 15% ), fractures (3/27, 11%), trauma (2/27, 7%), osteosarcoma (2/27, 7%), anterior cruciate ligament injury (1/27, 3.7%), acetabular roof lesions (1/27, 4%), and Perthes disease (1/27, 4%).

#### Social Media Platforms

In total, 35 articles were counted, of which 19 articles (54%) used or discussed a specific social media platform; 8 articles (23%) examined multiple platforms, specifying an average of 4.13 (SD 1.77) different platforms; and 8 articles (23%) did not specify a social media platform. Of those that specified a platform, the most common were YouTube (5/35, 14%), research websites (5/35, 14%), WeChat (3/35, 9%), Twitter (2/35, 6%), WhatsApp (2/35, 6%), Facebook (1/35, 3%), and Douyin (1/35, 3%).

#### Content for Social Media Use

Nearly half of the articles evaluated the quality of condition-specific video content (15/35, 43%). The remaining articles evaluated the use of social media for telemedicine (5/35, 14%), data collection via survey (3/35, 9%), and content retrieval, specifically assessing parental internet use (3/35, 9%). Table 3 lists all the contents of social media covered in the paper.

**Table 3:** Study characteristics (N=35).

Research subject			Study		Country of first author		Study design		Content		Social media platform		Condition
**Patients and parents**													
	Parents^a^	Bao et al [16], 2015		China		Cross-sectional		Retrieval survey		Not stated		AIS^b^	
	Patients^a^	Golden-Plotnik et al [17], 2018		United Kingdom		Randomized controlled trial		Health education		Websites		Fractures	
	Patients^a^	Kapıcıoğlu et al [18], 2019		Turkey		Retrospective		Telemedicine		WhatsApp		Trauma	
	Parents^a^	Peterlein et al [19], 2019		Germany		Cross-sectional		Retrieval survey		No stated		No specific	
	Patients^a^	Wang et al [20], 2020		China		Descriptive		Disease status survey		WeChat		ARLs^c^	
	Patients^a^	Yılmaz et al [21], 2023		Turkey		Quasi-experimental		Telemedicine		Websites		AIS	
	Patients^a^	Zhu et al [22], 2021		China		Quasi-experimental		Telemedicine		WeChat		AIS	
	Patients and parents^a^	Alsiddiky et al [23], 2019		Saudi Arabia		Cross-sectional		Cognitive survey		Not stated		Clubfoot	
	Patients and parents^d^	Donovan et al [24], 2021		Unites States		Semistructured interviews		Social support		YouTube, Facebook, and Twitter		Osteosarcoma	
	Patients and parents^a^	Gibbard et al [25], 2021		Canada		Cross-sectional study		Cognitive survey		Not stated		DDH^e^	
	Patients and parents^a^	Lysenko et al [26], 2016		Canada		Quasi-experimental		Retrieval survey		Not stated		AIS	
**Health researchers and practitioners**													
	Students^a^	Chen et al [27], 2023		China		Retrospective		Teaching		WeChat		No specific	
	Surgeons^d^	Chiang et al [28], 2022		United States		Cross-sectional		Physician media content evaluation		LinkedIn, Facebook, YouTube, Instagram, TikTok, Twitter, website, and ResearchGate		No specific	
	Surgeons^a^^,^^d^	Gibbard et al [29], 2021		Canada		Cross-sectional		Telemedicine		No stated		No specific	
	Surgeons^d^	Jay et al [30], 2021		United States		Descriptive		Physician evaluation		Websites		No specific	
	Surgeons^d^	Lander et al [9], 2017		United States		Cross-sectional		Physician media content evaluation		Facebook, Twitter, LinkedIn, ResearchGate, and YouTube		No specific	
**Public (recipients of information)**													
	Public	Alosaimi et al [31], 2022		Saudi Arabia		Cross-sectional		Cognitive survey		Not stated		Clubfoot	
**Social media related**													
	Accounts	Hanna et al [32], 2021		United States		Retrospective		Medical staff media survey		Facebook, Twitter, and YouTube		Clubfoot	
	Articles	Khalid et al [33], 2023		United States		Retrospective		Research		Twitter		No specific	
	Cases	Stahl et al [34], 2019		Israel		Quasi-experimental		Telemedicine		WhatsApp		Trauma	
	Comment	Para et al [35], 2021		United States		Cross-sectional		Content analysis		Facebook, Twitter, and YouTube		DDH	
	Postings	Truumees et al [36], 2021		United States		Cross-sectional		Content analysis		Facebook, Instagram, YouTube, and LinkedIn		AIS	
	Tweets	Kodali et al [37], 2021		Canada		Cross-sectional		Content analysis		Twitter		DDH	
	Videos	Cassidy et al [38], 2018		Ireland		Cross-sectional		Content analysis		YouTube		ACL^f^ injury	
	Videos	Kıvrak and Ulusoy [39], 2023		Turkey		Cross-sectional study (review)		Content analysis		YouTube		Fractures	
	Videos	Lock and Baker [40], 2022		New Zealand		Cross-sectional (review)		Content analysis		YouTube		DDH, SCFE^g^, and Perthes disease	
	Videos	Ranade et al [41], 2020		United States		Cross-sectional		Content analysis		YouTube		Clubfoot	
	Videos	Rudisill et al [42], 2023		United States		Cross-sectional		Content analysis		YouTube		AIS	
	Videos	Zhu et al [43], 2023		China		Observational		Content analysis		Douyin		Fractures	
	Websites	Fabricant et al [44], 2013		United States		Cross-sectional (review)		Content analysis		Websites		DDH	
	Websites	Heady et al [45], 2018		United States		Cross-sectional (review)		Content analysis		Websites		AIS	
	Websites	Lam et al [46], 2013		United States		Cross-sectional (review)		Content analysis		Facebook, Twitter, and YouTube		Osteosarcoma	
	Websites	Mc Carthy and Taylor [47], 2020		Ireland		Cross-sectional (review)		Content analysis		Facebook, Instagram, Tinder, and TikTok		DDH	
	Websites	Nassiri et al [48], 2015		Canada		Cross-sectional (review)		Content analysis		Not stated		Perthes disease	
	Websites	Ng et al [49], 2017		Ireland		Cross-sectional (review)		Content analysis		Facebook		AIS	

^a^Recipients of information.

^b^AIS: adolescent idiopathic scoliosis.

^c^ARL: acetabular roof lesion.

^d^Developers of information.

^e^DDH: developmental dysplasia of the hip.

^f^ACL: anterior cruciate ligament.

^g^SCFE: slipped capital femoral epiphysis.

#### Use of Social Media

Among the 35 articles, most used social media to help with medical staff and patient education and training (23/35, 66%) and to disseminate information (21/35, 60%), followed by helping medical staff collect data (8/35, 23%). Medical institutions and staff also used social media to increase attention (6/35, 17%), enhance social support (5/35,14%), facilitate the recruitment of research participants (3/35, 9%), support professional development (3/35, 9%), and implement health interventions (2/35, 5%). Table 4 illustrates the use of various applications.

**Table 4:** Social media use by social media platform.

Social media platform and condition		Purpose
**TikTok**		
	Fractures	Information dissemination and education and training
**Facebook**		
	AIS^a^	Information dissemination and education and training
**Twitter**		
	DDH^b^	Information dissemination and education and training
	Not specified	Professional development
**WeChat**		
	AIS	Data collection
	ARLs^c^	Data collection
	Not specified	Data collection
**WhatsApp**		
	Trauma	Data collection
**YouTube**		
	AIS	Information dissemination and education and training
	Clubfoot	Information dissemination and education and training
	Fractures	Information dissemination and education and training
	Anterior cruciate ligament injury	Information dissemination and education and training
	Multiple	Information dissemination and education and training
**Websites**		
	AIS	Health interventions, recruitment of research participants, information dissemination, education and training, increasing attention, and privacy ethics
	DDH	Information dissemination and education and training
	Fractures	Health interventions and education and training
	Not specified	Data collection and increasing attention
**Multiple platforms**		
	AIS	Information dissemination, education and training, social support, increasing attention, and professional development
	Clubfoot	Information dissemination, education and training, professional development, social support
	DDH	Increasing attention
	Osteosarcoma	Education and training, social support, and information dissemination
	Not specified	Information dissemination, education and training, increasing attention, and privacy ethics
**Not stated**		
	AIS	Information dissemination, education and training, and social support
	Clubfoot	Data collection
	DDH	Recruitment of study participants
	Pethes disease	Information dissemination and education and training
	Not specified	Recruitment of study participants and data collection

^a^AIS: adolescent idiopathic scoliosis.

^b^DDH: developmental dysplasia of the hip.

^c^ARL: acetabular roof lesion.

### The Types of Quality of Information Tools and Readability Tests

A total of 5 general quality of information (QOI) tools, 7 specific QOI tools, and 6 readability tools were used in the 12 studies under review. Table 5 provides an overview of these QOI tools and readability tests used in the studies.

**Table 5:** Overview of the QOI^a^ tools used in the studies.

QOI tools	Diseases	Description	Scoring
DISCERN [46,48,49]	—^b^	Comprises 16 questions, each scored on a 5-point Likert-type scale in relation to the completeness of the evaluated information.	Grade scoring: scores >63=excellent, 51-62=good, 39-50=fair, 27-38=poor, and 16-26=very poor
Modified DISCERN [38,39,41]	—	The modified DISCERN score evaluates clarity, reliability, bias/balance, provision of additional information sources, and areas of uncertainty.	Count scoring: 1 point is awarded for each criterion fulfilled, with resulting scores ranging from 0 to 5.
GQS^c^ [39,40,42,43]	—	GQS evaluates the educational value of online content for patients and their families.	Grade scoring: scores range from 1 to 5.
JAMA^d^ benchmarks [38,40-42,48]	—	Assess websites for the presence of 4 benchmarks within the websites.	Count scoring: the score ranges from 0 to 4.
HON^e^ seal [48]	—	Provided to websites that adhered to the HON Foundation’s Code of Conduct.	Authentication: websites satisfying the criteria could display a logo provided by the Foundation.
ACLSS^f^ [38]	ACL^g^	The ACLSS was adapted from the ACL-specific content score devised by Bruce-Brand et al to evaluate online information on ACL injury and reconstruction.	Count scoring and grade scoring: 1 point was assigned for each criterion, resulting in a maximum potential score of 25. Using the ACLSS, videos were categorized as very good (21-25), good (16-20), moderate (11-15), poor (6-10), and very poor (0-5).
Condition-specific scores [40]	DDH^h^, SCFE^i^, and Perthes disease	Condition-specific content scores were developed for DDH, SCFE, and Perthes disease to assess the educational quality of content related to each specific condition.	Count scoring: each answer is graded as yes=1 and no=0, with a total score out of 15. Higher scores indicate greater condition-specific educational quality.
Evaluation of online consumer health information for idiopathic scoliosis [45]	AIS^j^	Physicians review and evaluate the categorical information and score it.	Grade scoring: score the information on a scale of 1 (poor) to 5 (excellent) based on quality, accuracy, and completeness.
Perthes-specific content score [48]	Perthes disease	One point was allocated for each predefined term that was mentioned and related to the general aspects of the procedure, management options, and complications.	Count scoring: sites were scored from 0 to 25, with a score of 25 indicating the highest content quality.
PSS^k^ [42]	AIS	The scoring system was developed based on the guidelines published by the American Academy of Orthopaedic Surgeons [50] to evaluate information specific to pediatric scoliosis. The PSS scoring system is a 15-item assessment.	Count scoring: higher scores out of a maximum of 15 represent higher educational quality of pediatric scoliosis–specific content.
Quality and accuracy of online information about developmental hip dysplasia [44]	DDH	Using the information on the POSNA^l^ and AAOS^m^ websites as a gold standard for online information on the diagnosis and evaluation, treatment, and complications and outcomes of DDH, a content quality score was generated.	Count scoring: 1 point was awarded for each item, with a maximum score of 30.
SCSS^n^ [49]	AIS	The SCSS was previously described by Mathur et al [51] in 2005. The information presented on each site was reviewed, and the content was explored for 35 scoliosis-specific words.	Count scoring: on an ordinal scale with a maximum of 32 points, each website was awarded 1 point for mentioning each scoliosis-specific term.

^a^QOI: quality of information.

^b^Not applicable.

^c^GQS: global quality score.

^d^JAMA: *Journal of the American Medical Association*.

^e^HON: Health on the Net.

^f^ACLSS: anterior cruciate ligament–specific score.

^g^ACL: anterior cruciate ligament.

^h^DDH: developmental dysplasia of the hip.

^i^SCFE: slipped capital femoral epiphysis.

^j^AIS: adolescent idiopathic scoliosis.

^k^PSS: pediatric scoliosis score.

^l^POSNA: Pediatric Orthopaedic Society of North America.

^m^AAOS: American Academy of Orthopaedic Surgeons.

^n^SCSS: scoliosis content–specific score.

### Evaluation of QOI and Readability

#### Overview

All the 12 papers in which the study subject was a video or a website were analyzed for content quality, with the most common conditions of interest being DDH (3/12, 25%) and AIS (3/12, 25%), followed by Perthes disease (2/12, 17%) and fractures (2/12, 17%). The remaining conditions were acetabular roof lesions (1/12, 8%), osteosarcoma (1/12, 8%), and slipped capital femoral epiphysis (1/12, 8%). An analysis of the socia media platforms found that most studies chose YouTube (5/12, 42%) for the analysis of the quality of the content of the condition, followed by Facebook (3/12, 25%), websites (2/12, 17%), Douyin (1/12, 8%), and unstated social media platforms (1/12, 8%).

Each study provided, where possible, mean scores (mean), SDs, range values, and maximum values, which reflect the quality or readability of the online information. Table 6 summarizes the QOI and readability attributes according to the category of the information.

**Table 6:** Summary of the QOI^a^ and readability attributes according to category.

Study	Research subject, n	Content created by medical professionals (%)	QOI tools, n	QOI, mean (SD)	Readability tests, n	Readability tests, mean (SD)
Cassidy et al [38], 2018	39 videos	61.22	2 QOI tools; 1 specific QOI tool	Modified DISCERN (reviewer 1): 2.3 (SD 0.9) out of 4 Modified DISCERN (reviewer 2): 2.1 (SD 0.9) out of 4 JAMA^b^ (reviewer 1): 2.5 (SD 0.7) out of 4 JAMA (reviewer 2): 2.3 (SD 0.7) out of 4 ACLSS^c^ (reviewer 1): 6.3 (SD 3.5) out of 25 ACLSS (reviewer 2): 4.6 (SD 2.9) out of 25	—^d^	—
Kıvrak and Ulusoy [39], 2023	50 videos	94	2 QOI tools	GQS^e^: 3 (SD 1) out of 5 Modified DISCERN: 3 (SD 1) out of 4	—	—
Lock and Baker [40], 2022	120 videos (40 per condition)	56	2 QOI tools; 1 specific QOI tool	DDH^f^ JAMA: 2.18 (SD 0.87) out of 4 DDH GQS: 3.25 (SD 1.10) out of 5 DDH-specific score: 7.25 (3.27) out of 15 SCFE^g^ JAMA: 2.23 (SD 1.17) out of 4 SCFE GQS: 2.88 (SD 0.97) out of 5 SCFE specific score: 8.80 (SD 3.11) out of 15 Perthes disease JAMA: 2.20 (SD 0.91) out of 4 Perthes disease GQS 3 (SD 1.26) out of 5 Perthes disease–specific score: 8 (SD 3.57) out of 15	—	—
Ranade et al [41], 2020	42 videos	57	2 QOI tools	Modified DISCERN: 2.1 (SD 1.07); range 0.3-4 out of 4 JAMA: 0.9 (SD 0.65); range 0-2 out of 4	—	—
Rudisill et al [42], 2023	153 videos	73.20	2 QOI tools; 1 specific QOI tool	JAMA: 1.3 (SD 0.7); range 0-3 out of 4 GQS: 1.7 (SD 0.8); range 1-4 out of 5 PSS^h^: 6.2 (SD 3.6); range 0-14 out of 15	—	—
Zhu et al [43], 2023	74 videos	52.70	1 QOI tool	GQS: 3; range 1-5 out of 5	—	—
Fabricant et al [44], 2013	63 websites	—	1 specific QOI tool	Quality: 18.8 (SD 5.7); range 5-28 out of 30 Accuracy: 10.7 (SD 1.8); range 3-12 out of 12	1	FKGL^i^: 0.5 (SD 2.4); range 5-17
Heady et al [45], 2018	10 websites		1 specific QOI tool	Evaluation of online consumer health information for idiopathic scoliosis: 47.63; range 15.67-62.67 out of 75	1	FRES^j^: 48.75
Lam et al [46], 2013	56 websites		1 QOI tool	DISCERN: 49.8; range 31-66 out of 80	1	FRES: 44.5 (range 16.8-66)
Mc Carthy and Taylor [47], 2020	21 websites	76.19	—	—	6	FRES: 52.55 (SD 15.43) FKGL: 7.87 (SD 2.04) GFI^k^: 8.60 (SD 2.49) SMOG^l^: 7.03 (SD 1.84) ARI^m^: 6.38 (SD 2.83) CLI^n^: 13.97 (SD 3.02) RGL^o^: 8.67 (SD 1.82)
Nassiri et al [48], 2015	45 websites	88.89	3 QOI tools; 1 specific QOI tool	DISCERN: 53.1 (SD 9); range 30-70 out of 80 JAMA: 2.1 (SD 1.2); range 0-4 out of 4 Perthes-specific content: 15.8 (SD 4.1); range 5-23 out of 25 HON^p^ seal: 3 websites out of 45 websites	—	—
Ng et al [49], 2017	86 websites	—	1 QOI tool; 1 specific QOI tool	DISCERN: 22.5 (SD 7.6); range 16-45 out of 80 SCSS^q^: 5.7 (SD 5.8); range 0-20 out of 32	—	—

^a^QOI: quality of information.

^b^JAMA: *Journal of the American Medical Association*.

^c^ACLSS: anterior cruciate ligament injury–specific score.

^d^Not applicable.

^e^GQS: global quality score.

^f^DDH: developmental dysplasia of the hip.

^g^SCFE: slipped capital femoral epiphysis.

^h^PSS: pediatric scoliosis–specific.

^i^FKGL: Flesch-Kincaid Grade Level.

^j^FRES: Flesch-Reading Ease Score.

^k^GFI: Gunning Fog index.

^l^SMOG: Simple Measure of Gobbledygook.

^m^ARI: automated readability index.

^n^CLI: Coleman-Liau index.

^o^RGL: reading grade level.

^p^HON: Health on the Net Foundation.

^q^SCSS: scoliosis-specific content score.

#### General QOI Tools Analysis for Quality Evaluation

Of the 9 studies, 1 (11%) study used 3 general QOI tools, while 5 (56%) studies used 2 general QOI tools, and 3 (33%) studies exclusively applied 1 general QOI tool. The *Journal of the American Medical Association* (JAMA) benchmarks were the most commonly used QOI tools in this review—each used in 56% (5/9) of the studies. According to the global quality score (GQS) ratings, of the 4 (44%) studies that used the GQS evaluation tool, 1 (25%) rated the quality and flow as good (rated 4 out of 5), 2 (50%) rated them as suboptimal (rated 3 out of 5), and 1 (25%) rated them as poor (rated 2 out of 5). The DISCERN and modified DISCERN tools were used in 3 (33%) of the studies. According to the DISCERN ratings, of the 3 studies, 1 (33%) was rated as good (mean score range: 39-50), 1 (33%) as fair (mean score range: 27-38), and 1 (33%) as poor (mean score range: 16-26). The Health on the Net (HON) Code seal was used in only 1 (11%) of the studies.

#### Specific QOI Tools Analysis for Quality Evaluation

A total of 7 specific QOI tools were used in that article, indicating that these tools have not been widely disseminated or adopted by other studies. Of the 7 studies [38,40,42,44,45,48,49] using the special QOI tools, 1 (14%) evaluated the quality as good, 4 (57%) as fair, and 2 (29%) as poor. There was an inconsistency in the study by Cassidy et al [38], in which the general QOI tools evaluated the results as fair, whereas the anterior cruciate ligament injury–specific score rated it as poor. Of the special QOI tools used, 3 (43%) were applied to AIS, 2 (29%) to Perthes disease and DDH, and 1 (14%) to slipped capital femoral epiphysis and anterior cruciate ligament injury.

#### Readability Tests Analysis for Readability Evaluation

A total of 4 studies included the readability assessments of health information. Only 1 (25%) study used 6 readability tools, whereas the remaining 3 (75%) used only 1 readability tool. The Flesch Reading Ease Score (FRES), which indicates acceptable readability with a score of ≥65, was the most frequently used tool, but none of the 3 studies in which it was applied achieved this threshold. According to the evaluation scores, the readability of health information appears to be at a college reading level, as determined by the FRES school-level scale. The results of the readability test indicate that some of the health information is difficult to read and may not be easily understood by all patients. Multimedia Appendix 3 lists the readability tests used in the studies.

## Discussion

### Principal Findings

#### Overview

Through this scoping review, we summarized the current state of research in the existing literature on the use of social media in pediatric orthopedics, in 35 articles that met the inclusion criteria, published between 2013 and 2023. Our study found that there has been an increase in the number of studies on the use of social media in pediatric orthopedics over the past decade, particularly in the past 5 years. This trend may reflect an increase in research interest in the field; however, it is important to note that because of the limited sample size, this finding still needs to be further confirmed by a wider range of studies. This suggests that more people are focusing on pediatric orthopedic conditions on social media, and the number of papers published on social media is likely to continue increasing. Research on social media has largely focused on the United States, which is consistent with statistics on global social media penetration. We identified the 3 main social media platforms used for pediatric orthopedic conditions (covering multiple social media platforms) as YouTube, Twitter, and Facebook, with the other platforms being relatively less used, possibly because of geographical factors. The most studied diseases were AIS, DDH, and clubfoot, which are also common pediatric orthopedic conditions with a higher prevalence than other chronic conditions. A large portion of the included studies, all of which were cross-sectional studies, were based on videos and websites and analyzed the quality of content.

#### Types of Social Media Used

We applied the SMILE [14] framework to analyze the resulting types of social media applications, which include data collection, dissemination of information, education and training, health interventions, increasing attention, privacy ethics, professional development, recruitment of study participants, and social support. The importance of social media in health care information dissemination, education, training, and patient support has been statistically demonstrated, highlighting the extensive influence of social media in modern health care.

#### Information Dissemination

Many studies identified social media as a powerful tool for rapidly and widely disseminating health information. This role became especially prominent during the COVID-19 pandemic [28]. Medical institutions, hospitals, and communities often use platforms such as Facebook, Twitter, and LinkedIn to share accurate and timely information. These official channels are generally viewed as trustworthy and play a key role in countering misinformation [52], especially in areas involving rare pediatric conditions [53], while also making orthopedic research content more persuasive and engaging, with broad applicability to related scientific fields. Social media also facilitates the dissemination of research findings and enhances public engagement [54]. Despite these advantages, some health care professionals still underuse social media for proactive patient engagement [36].

#### Health Intervention

Various medical institutions can implement health interventions with the help of social media. The health resources provided include delivering health information to audiences [55], motivating participation in health-related activities [56], increasing physical activity and medical screening tests [57], and guiding sports. Yılmaz et al [21] used the Scoliosis Tele-Screening Test tool to help parents assess whether their children were at risk for scoliosis without needing to visit a medical facility for a checkup, amid the suspension of schooling and education owing to the COVID-19 outbreak. With limited access to health professionals, interest in telemedicine and virtual examinations increased significantly. The research team developed this tool in response to the question of how parents might identify possible scoliosis. In general, research shows that health education via social media can transcend geographical and time constraints and is characterized by wide coverage, strong practicability, timeliness, convenience, and efficiency. Children and their families may accept the integration of health interventions into the media and effectively increase health knowledge, reduce risks, and guide healthy behaviors.

#### Education and Training

Social media also serves as a platform for education, helping patients and families understand condition processes, treatment options, and self-care strategies [8,58]. For example, some parents sought timely medical attention after watching informative online videos [39,44], illustrating the value of digital health education. In professional education, social media supports flexible, interactive learning for medical students and clinicians. It enhances engagement, reduces stress, and complements traditional instructional methods [27,59]. However, the presence of misleading content remains a challenge, highlighting the need for professional oversight [60].

#### Social Support

The emergence of social media has provided the public with broad communication opportunities and has helped create a social support space for parents of children with conditions. This support mainly includes informational support, emotional support, respect support, and network support [61], which is conducive to reducing the psychological anxiety of caregivers. Family members of children with the same condition can work together on social media platforms to create online‑based support groups and communities, improve health literacy [62,63], share diagnosis and treatment experiences, encourage and support each other, and foster a sense of belonging and identity [64].

#### Increasing Attention

Beyond communication and education, social media is used strategically to raise awareness of conditions and promote institutional visibility. Medical professionals use platforms such as Twitter, YouTube, and TikTok to increase their public presence and build reputations [9,28,45]. Online feedback plays a significant role in shaping public perception—positive reviews can boost appointment rates, while negative ones may deter service use, especially in high–information asymmetry settings, such as health care [65]. This underscores the importance of active reputation management in digital spaces.

#### Recruitment of Study Participants

Online recruitment methods are characterized by anonymity and discretion. For sensitive topics, online questionnaires can reduce the feelings of shame and elicit more honest responses [66]. They also enable the recruitment of more qualified participants, reducing the time and cost of recruitment [67], and they are conducive for research involving rare groups [68]. However, there may be ethical issues in social media recruitment. Social media users have become accustomed to “consent” and may not carefully read the study’s fine print, which limits the effectiveness of informed consent [69]. Researchers and participants should carefully design studies to avoid ethical flaws, critically assess potential selection bias, and add reminders promptly to ensure the rigor of the research and safeguard the interests of participating patients.

#### Professional Development

The internet and social media have promoted the development of medical journals, such as *Arthroscopy*, *Arthroscopy Technology*, and *Arthroscopy, Sports Medicine and Rehabilitation* [70]. Numerous medical professionals use Twitter to disseminate published and unpublished research quickly. Social media serves as a platform for advancement, collaboration beyond traditional boundaries, and public health education [71].

### Assessment Tools for QOI and Readability of Information

Regarding the tools for assessing the quality of social media content about pediatric orthopedic conditions, researchers often use DISCERN, modified DISCERN, JAMA, GQS, and HONcode, which have generally been shown to be effective in assessing the QOI. However, DISCERN, JAMA, and HONcode apply to websites and were not developed specifically for video content. Therefore, the applicability of these tools needs to be explored, and it should be noted that the DISCERN tool was originally designed for written content. Because of the rigor of the questions, when applied to video-based content, the category scores may be lower [72], and therefore, researchers may not be able to assess QOI using a single tool accurately.

Specific QOI tools are diverse and can effectively evaluate quality based on condition, but there are differences in variables and evaluation criteria, and the evaluation results of different studies lack comparability to some extent. Some specific QOI tools only have entry scores and lack rating criteria, so they cannot qualitatively judge the QOI. The review found that the specific QOI tools were not effectively disseminated, and different specific QOI tools were used for the same category of AIS diseases, probably because the researchers did not realize that they could retrieve the tools that had already been developed at the time of the study, or that the specific QOI tools for a particular condition could not be widely used in other situations.

Only 4 studies [44-47] in pediatric orthopedic conditions focused on the readability of health information on social media, suggesting that researchers are not paying enough attention to this aspect. However, the formula calculation may be difficult for some researchers. Readability is important for patients, and more difficult information may not be understood by patients, or may even be misinterpreted, which is not conducive to doctor-patient communication and leads to an increased burden on medical staff. In addition, better readability makes it easier for the public to choose to watch and enhances the duration of viewing.

### Accuracy and Reliability of Pediatric Orthopedics Information in Social Media

Overall, there is a small amount of literature assessing the quality of short social media videos related to pediatric orthopedic conditions and health sciences. We found a limited number of publications (n=12), and our results suggested that most pediatric orthopedic condition–related information is of moderate quality. Some studies have suggested that physicians recommend sites with the HONcode logo as an indicator of reliable information or sites they have personally reviewed. In addition, social media platform searches should be upgraded with the appropriate algorithms to recommend and encourage high-quality pediatric orthopedic health education information. This requires a concerted effort by health care providers and medical staff to maximize the benefits of social media.

According to most studies analyzed [40-42], information provided by a large proportion of medical staff and professional organizations usually has higher quality scores. The explanations of illnesses by nonprofessionals are of poor quality and may even be misleading, ultimately affecting the doctor-patient relationship. The proportion of non–health care professionals was maintained at 3%, which would be more favorable for video viewers to be exposed to high-quality content, considering that the proportion of non–health care professionals was maintained at 3% [39].

Most of the evaluated websites had a FRES result indicating a university-graduate reading level, which is higher than the recommended standard [44]. Some accurate and reliable health information content is difficult for patients to understand and contains many medical terms that are difficult to read; therefore, there is a need to improve readability and provide translation services, which will improve the usability of social media. This suggests that health care organizations and health care professionals need to consider the literacy level of the target audience when publishing professional health information content, which can be done using diagrams or animated videos in educational materials to convey meaning, using simple vocabulary in a conversational style and avoiding jargon.

### Future Expectations

Currently, the development of medical social media is still in its preliminary stage. There are problems, such as uncertainties related to internet-obtained information, individual differences in use, lack of conditions for interdisciplinary teamwork, and insufficient development of the institutional system [73]. The support of the national government, society, medical institutions, medical personnel, and the public is essential. This is necessary to promote the equalization of basic public services in the health field; maintain the public welfare nature of basic medical and health services; gradually narrow the differences in basic health services and health levels between urban and rural areas, regions, and groups; achieve universal health coverage; and promote social equity.

Within the current economic, medical, and educational environment, the effect of social media–based science education is becoming increasingly significant. At present, social media is mostly oriented toward diagnosed diseases and focuses on reducing the occurrence of complications and promoting tertiary prevention in disease management. It is helpful for disease treatment, prognosis, and recovery, and reduces the disability and mortality rates of children. Development of this field indicates that the future aim is to promote disease screening to achieve the secondary prevention goals of early detection and early treatment, improving the public’s ability to identify diseases, and preventing patients from missing the best treatment opportunities. The effect of primary prevention is to develop healthy living habits and exercise methods. We should focus on mastering disease prevention knowledge, integrating internet-based medical care with health examinations and health consultations, promoting the development of health services, and realizing the construction of a health innovation network.

Telemedicine is also gradually gaining public attention. Using social media platforms, online consultations can be achieved without leaving home. Although current social media is still limited to text, pictures, or videos, new diagnoses and treatment models can be created based on scientific and technological progress. With the help of various social media platforms, data regarding children’s current health conditions are collected through questionnaires and other forms and assessed and analyzed by medical professionals in the telemedicine system. Then, based on the classification of children’s health status, the system determines whether further observation or health intervention is needed and integrates this information to form an overview of the child’s health status and provide parents with professional advice, for example, the risk of pediatric orthopedics in the future and what they need to pay attention to. Science popularization has targeted health knowledge and improved parents’ ability to identify conditions. By retaining data after testing, the system can make personalized recommendations based on various aspects of children’s physical conditions, deliver precise health education, and protect children’s health in many aspects, such as diet, exercise, and psychology, and ensure healthy physical and mental growth of children. Hospitals and communities establish relevant databases to form an all-in-one care model of medical treatment, nursing, nutrition, and rehabilitation.

### Limitations

This study has certain limitations. Because of the limited databases searched, some relevant articles may have been omitted. Conference papers, dissertations, and gray literature were also excluded. In addition, we searched only for keywords found in the title and abstract, rather than the full text, which may have resulted in the exclusion of articles that mentioned social media in pediatric orthopedics in the review. Furthermore, most of the included articles evaluated content created by health care providers, offering limited information on the quality, accuracy, and readability of content created by the public.

### Conclusions

This scoping review found that literature on the use of social media in pediatric orthopedic conditions is steadily increasing, and that social media plays an increasingly important role in knowledge dissemination and educational engagement. A variety of tools have been used to assess the QOI, but partly differences in tool types limit comparability across studies. In addition, only a small number of studies have evaluated the readability of online content, leaving this area underexplored. Overall, the quality of pediatric orthopedic information available on social media is generally fair, with readability levels often exceeding recommended standards. Health care professionals are encouraged to actively participate in social media by providing accurate, easy-to-understand content evaluated using reliable metrics to support patient education and informed decision-making. Future research should continue to investigate the role of social media in pediatric orthopedics, with particular attention to improving both the quality and accessibility of information.

## Appendix

Multimedia Appendix 1 Search strategy.

Multimedia Appendix 2 PRISMA-ScR checklist.

Multimedia Appendix 3 Overview of the readability tests used in the studies.

## Abbreviations

AIS: adolescent idiopathic scoliosis
DDH: Developmental Dysplasia of the Hip
FRES: Flesch Reading Ease Score
GQS: global quality score
HON: Health on the Net
JAMA: *Journal of the American Medical Association*
PRISMA-ScR: Preferred Reporting Items for Systematic Reviews and Meta-Analyses Extension for Scoping Reviews
QOI: quality of information
SMILE: Social Media for Implementing Evidence
