# Supplementary material for: Applications and Assessment of Social Media in Pediatric Orthopedics: Scoping Review
Source: J Med Internet Res. 2025 Aug 14;27:e55360. doi: 10.2196/55360 (PMC12395100; doi:10.2196/55360)
Supplement: Multimedia Appendix 1 [file jmir_v27i1e55360_app1.docx]

| Concepts | Pubmed search strategy | Research | Results |
| --- | --- | --- | --- |
| Pediatric (controlled vocabulary) | "adolescent"[MeSH] OR "Child"[Mesh] OR "Infant"[Mesh] OR "Pediatrics"[Mesh] | #1 | 3,998,413 |
| Pediatric (free text) | adolescen*[TIAB] OR baby[TIAB] OR babies*[TIAB] OR boy[TIAB] OR boys[TIAB] OR toddler[TIAB] OR preschool*[TIAB] OR child*[TIAB] OR girl*[TIAB] OR infan*[TIAB] OR kid[TIAB] OR kids[TIAB] OR neonat*[TIAB] OR newborn*[TIAB] OR paediatric*[TIAB] OR pediatric*[TIAB] | #2 | 2,801,493 |
| Total for Pediatric | #1 OR #2 | #3 | 4,790,230 |
| Social media (controlled vocabulary) | "Social Networking"[Mesh] OR "Social Media"[Mesh] | #4 | 20,960 |
| Social media (free text) | New media[TIAB] OR Networking, Social[TIAB] OR Social Networks[TIAB] OR Network, Social[TIAB] OR Social Network[TIAB] OR Social media[TIAB] OR Digital media[TIAB] OR Instagram[TIAB] OR internet media[TIAB] OR internet medium[TIAB] OR media of the network[TIAB] OR network media[TIAB] OR network medium[TIAB] OR online media[TIAB] OR facebook[TIAB] OR google[TIAB] OR youtube[TIAB] OR whatsapp[TIAB] OR tumblr[TIAB] OR twitter[TIAB] OR linkedin[TIAB] OR instagram[TIAB] OR pinterest[TIAB] OR snapchat[TIAB] OR reddit[TIAB] OR qzone[TIAB] OR weibo[TIAB] OR wechat[TIAB] OR youku[TIAB] OR tudou[TIAB] OR renren[TIAB] OR badoo[TIAB] OR baidu[TIAB] OR orkut[TIAB] OR foursquare[TIAB] OR vine[TIAB] OR vkontakte[TIAB] OR myspace[TIAB] OR hi5[TIAB] OR tiktok[TIAB] OR tik tok[TIAB] OR bebo[TIAB] | #5 | 112,587 |
| Total for Social media | #4 OR #5 | #6 | 117,018 |
| Musculoskeletal Diseases(controlled vocabulary) | "Genu Varum"[Mesh] OR "Equinus Deformity"[Mesh] OR "Flatfoot"[Mesh] OR "Hip Dislocation, Congenital"[Mesh] OR "Polydactyly"[Mesh] OR "Scoliosis"[Mesh] OR "Skeleton"[Mesh] OR "Bone and Bones"[Mesh] OR "orthopedics"[Mesh] OR "Musculoskeletal Diseases"[Mesh] | #7 | 1,736,821 |
| Musculoskeletal Diseases(free text) | Equinus Deformity[TIAB] OR Genu Varum[TIAB] OR Flatfoot[TIAB] OR Polydactyly[TIAB] OR Scoliosis[TIAB] OR Hip Dislocation, Congenital[TIAB] OR Bow Leg[TIAB] OR Bow Legs[TIAB] OR Leg, Bow[TIAB] OR Legs, Bow[TIAB] OR Genu Varus[TIAB] OR Deformities, Equinus[TIAB] OR Deformity, Equinus[TIAB] OR Equinus Deformities[TIAB] OR Talipes Equinus[TIAB] OR Equinus Contracture[TIAB] OR Contractures, Equinus[TIAB] OR Contracture, Equinus[TIAB] OR Equinus Contractures[TIAB] OR Talipes Equinovalgus[TIAB] OR Equinovalgus, Talipes[TIAB] OR Talipes Valgus[TIAB] OR Valgus, Talipes[TIAB] OR Splayfoot[TIAB] OR Flat Feet[TIAB] OR Feet, Flat[TIAB] OR Flat Foot[TIAB] OR Pes Planus[TIAB] OR Foot, Flat[TIAB] OR Flatfeet[TIAB] OR Vertical Talus[TIAB] OR Rigid Flatfoot[TIAB] OR Talus, Vertical[TIAB] OR Rocker-Bottom Foot[TIAB] OR Flatfoot, Rigid[TIAB] OR Foot, Rocker-Bottom[TIAB] OR Rocker Bottom Foot[TIAB] OR Pes Valgus, Congenital Convex[TIAB] OR Vertical Talus, Congenital[TIAB] OR Congenital Vertical Talus[TIAB] OR Talus, Congenital Vertical[TIAB] OR Convex Pes Valgus[TIAB] OR Pes Valgus, Convex[TIAB] OR Foot, Convex[TIAB] OR Convex Foot[TIAB] OR Talipes Calcaneovalgus[TIAB] OR Calcaneovalgus, Talipes[TIAB] OR Acquired Adult Flatfoot DefORmity[TIAB] OR Flexible Flatfoot[TIAB] OR Flatfoot, Flexible[TIAB] OR Polydactylies[TIAB] OR Polydactylia[TIAB] OR Hyperdactyly[TIAB] OR Congenital Hip Dislocations[TIAB] OR Dislocations, Congenital Hip[TIAB] OR Hip Dislocations, Congenital[TIAB] OR Congenital Hip Dislocation[TIAB] OR Congenital Hip Displacement[TIAB] OR Displacements, Congenital Hip[TIAB] OR Congenital Hip Displacements[TIAB] OR Hip Displacements, Congenital[TIAB] OR Congenital Hip Dysplasia[TIAB] OR Congenital Hip Dysplasias[TIAB] OR Hip Dysplasias, Congenital[TIAB] OR Dysplasias, Congenital Hip[TIAB] OR Dislocation, Congenital Hip[TIAB] OR Hip, Dislocation Of, Congenital[TIAB] OR Displacement, Congenital Hip[TIAB] OR Dysplasia, Congenital Hip[TIAB] OR Hip Displacement, Congenital[TIAB] OR Hip Dysplasia, Congenital[TIAB] OR Congenital Dysplasia Of The Hip[TIAB] OR Dislocation Of Hip, Congenital[TIAB] OR Hip Dysplasia, Congenital, Nonsyndromic[TIAB] OR skeleton[TIAB] OR bones[TIAB] OR osteopathic[TIAB] OR orthopaedic[TIAB] OR child osteology[TIAB] OR musculoskeletal disease[TIAB] OR orthopedic disorders[TIAB] OR orthopedic disorder[TIAB] OR musculoskeletal symptom*[TIAB] OR musculoskeletal pain[TIAB] OR musculoskeletal complaint*[TIAB] OR orthopedic injur*[TIAB] OR orthopaedic injur*[TIAB] OR deformity[TIAB] | #8 | 265,136 |
| Total for Musculoskeletal Diseases | #7 OR #8 | #9 | 1,851,240 |
| Overall | #3 AND #6 AND #9 | #10 | 505 |

| Concepts | Web of Science search strategy | Research | Results |
| --- | --- | --- | --- |
| Pediatric | TI=(adolescen* OR baby OR babies* OR boy OR boys OR toddler OR preschool* OR child* OR girl* OR infan* OR kid OR kids OR neonat* OR newborn* OR paediatric* OR pediatric*) and Preprint Citation Index (Exclude – Database) | #1 | 2279863 |
|  | AB=(adolescen* OR baby OR babies* OR boy OR boys OR toddler OR preschool* OR child* OR girl* OR infan* OR kid OR kids OR neonat* OR newborn* OR paediatric* OR pediatric*) and Preprint Citation Index (Exclude – Database) | #2 | 2921447 |
|  | #1 OR #2 and Preprint Citation Index (Exclude – Database) | #3 | 3752306 |
| Social media | TI=(New media OR Social Networking OR Social Networks OR Network, Social OR Social Network OR Social media OR Digital media OR Instagram OR internet media OR internet medium OR media of the network OR network media OR network medium OR online media OR Facebook OR Google OR youtube OR WhatsApp OR Tumblr OR Twitter OR linkedin OR Instagram OR Pinterest OR Snapchat OR reddit OR qzone OR weibo OR WeChat OR youku OR tudou OR Renren OR badoo OR Baidu OR orkut OR foursquare OR vine OR Vkontakte OR myspace OR hi5 OR tiktok OR tik tok OR bebo) and Preprint Citation Index (Exclude – Database) | #4 | 85386 |
|  | AB=(New media OR Social Networking OR Social Networks OR Network, Social OR Social Network OR Social media OR Digital media OR Instagram OR internet media OR internet medium OR media of the network OR network media OR network medium OR online media OR Facebook OR Google OR youtube OR WhatsApp OR Tumblr OR Twitter OR linkedin OR Instagram OR Pinterest OR Snapchat OR reddit OR qzone OR weibo OR WeChat OR youku OR tudou OR Renren OR badoo OR Baidu OR orkut OR foursquare OR vine OR Vkontakte OR myspace OR hi5 OR tiktok OR tik tok OR bebo) and Preprint Citation Index (Exclude – Database) | #5 | 630305 |
|  | #4 OR #5 and Preprint Citation Index (Exclude – Database) | #6 | 649741 |
| Musculoskeletal Diseases | TI=(Equinus Deformity OR Genu Varum OR Flatfoot OR Polydactyly OR Scoliosis OR Hip Dislocation, Congenital OR Bow Leg OR Bow Legs OR Leg, Bow OR Legs, Bow OR Genu Varus OR Deformities, Equinus OR Deformity, Equinus OR Equinus Deformities OR Talipes Equinus OR Equinus Contracture OR Contractures, Equinus OR Contracture, Equinus OR Equinus Contractures OR Talipes Equinovalgus OR Equinovalgus, Talipes OR Talipes Valgus OR Valgus, Talipes OR Splayfoot OR Flat Feet OR Feet, Flat OR Flat Foot OR Pes Planus OR Foot, Flat OR Flatfeet OR Vertical Talus OR Rigid Flatfoot OR Talus, Vertical OR Rocker-Bottom Foot OR Flatfoot, Rigid OR Foot, Rocker-Bottom OR Rocker Bottom Foot OR Pes Valgus, Congenital Convex OR Vertical Talus, Congenital OR Congenital Vertical Talus OR Talus, Congenital Vertical OR Convex Pes Valgus OR Pes Valgus, Convex OR Foot, Convex OR Convex Foot OR Talipes Calcaneovalgus OR Calcaneovalgus, Talipes OR Acquired Adult Flatfoot Deformity OR Flexible Flatfoot OR Flatfoot, Flexible OR Polydactylies OR Polydactylia OR Hyperdactyly OR Congenital Hip Dislocations OR Dislocations, Congenital Hip OR Hip Dislocations, Congenital OR Congenital Hip Dislocation OR Congenital Hip Displacement OR Displacements, Congenital Hip OR Congenital Hip Displacements OR Hip Displacements, Congenital OR Congenital Hip Dysplasia OR Congenital Hip Dysplasias OR Hip Dysplasias, Congenital OR Dysplasias, Congenital Hip OR Dislocation, Congenital Hip OR Hip, Dislocation Of, Congenital OR Displacement, Congenital Hip OR Dysplasia, Congenital Hip OR Hip Displacement, Congenital OR Hip Dysplasia, Congenital OR Congenital Dysplasia Of The Hip OR Dislocation Of Hip, Congenital OR Hip Dysplasia, Congenital, Nonsyndromic OR skeleton OR bones OR osteopathic OR orthopaedic OR child osteology OR musculoskeletal disease OR orthopedic disorders OR orthopedic disorder OR musculoskeletal symptom* OR musculoskeletal pain OR musculoskeletal complaint* OR orthopedic injur* OR orthopaedic injur* OR deformity) and Preprint Citation Index (Exclude – Database) | #7 | 491173 |
|  | AB=(Equinus Deformity OR Genu Varum OR Flatfoot OR Polydactyly OR Scoliosis OR Hip Dislocation, Congenital OR Bow Leg OR Bow Legs OR Leg, Bow OR Legs, Bow OR Genu Varus OR Deformities, Equinus OR Deformity, Equinus OR Equinus Deformities OR Talipes Equinus OR Equinus Contracture OR Contractures, Equinus OR Contracture, Equinus OR Equinus Contractures OR Talipes Equinovalgus OR Equinovalgus, Talipes OR Talipes Valgus OR Valgus, Talipes OR Splayfoot OR Flat Feet OR Feet, Flat OR Flat Foot OR Pes Planus OR Foot, Flat OR Flatfeet OR Vertical Talus OR Rigid Flatfoot OR Talus, Vertical OR Rocker-Bottom Foot OR Flatfoot, Rigid OR Foot, Rocker-Bottom OR Rocker Bottom Foot OR Pes Valgus, Congenital Convex OR Vertical Talus, Congenital OR Congenital Vertical Talus OR Talus, Congenital Vertical OR Convex Pes Valgus OR Pes Valgus, Convex OR Foot, Convex OR Convex Foot OR Talipes Calcaneovalgus OR Calcaneovalgus, Talipes OR Acquired Adult Flatfoot Deformity OR Flexible Flatfoot OR Flatfoot, Flexible OR Polydactylies OR Polydactylia OR Hyperdactyly OR Congenital Hip Dislocations OR Dislocations, Congenital Hip OR Hip Dislocations, Congenital OR Congenital Hip Dislocation OR Congenital Hip Displacement OR Displacements, Congenital Hip OR Congenital Hip Displacements OR Hip Displacements, Congenital OR Congenital Hip Dysplasia OR Congenital Hip Dysplasias OR Hip Dysplasias, Congenital OR Dysplasias, Congenital Hip OR Dislocation, Congenital Hip OR Hip, Dislocation Of, Congenital OR Displacement, Congenital Hip OR Dysplasia, Congenital Hip OR Hip Displacement, Congenital OR Hip Dysplasia, Congenital OR Congenital Dysplasia Of The Hip OR Dislocation Of Hip, Congenital OR Hip Dysplasia, Congenital, Nonsyndromic OR skeleton OR bones OR osteopathic OR orthopaedic OR child osteology OR musculoskeletal disease OR orthopedic disorders OR orthopedic disorder OR musculoskeletal symptom* OR musculoskeletal pain OR musculoskeletal complaint* OR orthopedic injur* OR orthopaedic injur* OR deformity) and Preprint Citation Index (Exclude – Database) | #8 | 1166564 |
|  | #7 OR #8 and Preprint Citation Index (Exclude – Database) | #9 | 1335804 |
| Overall | #3 AND #6 AND #9 and Preprint Citation Index (Exclude – Database) | #10 | 699 |

| Concepts | Embase search strategy | Research | Results |
| --- | --- | --- | --- |
| Pediatric (controlled vocabulary) | 'child'/exp OR 'adolescent'/exp OR 'infant'/exp OR 'pediatrics'/exp | #1 | 4367738 |
| Pediatric (free text) | adolescen*:ti,ab OR baby:ti,ab OR babies*:ti,ab OR boy:ti,ab OR boys:ti,ab OR toddler:ti,ab OR preschool*:ti,ab OR child*:ti,ab OR girl*:ti,ab OR infan*:ti,ab OR kid:ti,ab OR kids:ti,ab OR neonat*:ti,ab OR newborn*:ti,ab OR paediatric*:ti,ab OR pediatric*:ti,ab | #2 | 3612442 |
| Total for Pediatric | #1 OR #2 | #3 | 6076246 |
| Social media (controlled vocabulary) | 'social network'/exp OR 'social media'/exp | #4 | 70784 |
| Social media (free text) | 'new media':ti,ab OR 'social networking':ti,ab OR 'social networks':ti,ab OR network,social:ti,ab OR 'social network':ti,ab OR 'social media':ti,ab OR 'digital media':ti,ab OR 'internet media':ti,ab OR 'internet medium':ti,ab OR 'media of the network':ti,ab OR 'network media':ti,ab OR 'network medium':ti,ab OR 'online media':ti,ab OR facebook:ti,ab OR google:ti,ab OR youtube:ti,ab OR whatsapp:ti,ab OR tumblr:ti,ab OR twitter:ti,ab OR linkedin:ti,ab OR instagram:ti,ab OR pinterest:ti,ab OR snapchat:ti,ab OR reddit:ti,ab OR qzone:ti,ab OR weibo:ti,ab OR wechat:ti,ab OR youku:ti,ab OR tudou:ti,ab OR renren:ti,ab OR badoo:ti,ab OR baidu:ti,ab OR orkut:ti,ab OR foursquare:ti,ab OR vine:ti,ab OR vkontakte:ti,ab OR myspace:ti,ab OR hi5:ti,ab OR tiktok:ti,ab OR 'tik tok':ti,ab OR bebo:ti,ab | #5 | 137266 |
| Total for New media | #4 OR #5 | #6 | 158196 |
| Musculoskeletal Diseases(controlled vocabulary) | 'varus knee'/exp OR 'varus knee' OR 'pes equinus'/exp OR 'pes equinus' OR 'flatfoot'/exp OR 'flatfoot' OR 'congenital hip dislocation'/exp OR 'congenital hip dislocation' OR 'polydactyly'/exp OR 'polydactyly' OR 'scoliosis'/exp OR 'scoliosis' OR 'bone'/exp OR 'bone' OR 'skeleton'/exp OR 'skeleton' OR 'orthopedics'/exp OR 'orthopedics' OR 'musculoskeletal disease'/exp | #7 | 4235266 |
| Musculoskeletal Diseases(free text) | 'equinus deformity':ti,ab OR 'genu varum':ti,ab OR flatfoot:ti,ab OR polydactyly:ti,ab OR scoliosis:ti,ab OR 'hip dislocation,congenital':ti,ab OR 'bow leg':ti,ab OR 'bow legs':ti,ab OR leg,bow:ti,ab OR legs,bow:ti,ab OR 'genu varus':ti,ab OR deformities,equinus:ti,ab OR deformity,equinus:ti,ab OR 'equinus deformities':ti,ab OR 'talipes equinus':ti,ab OR 'equinus contracture':ti,ab OR contractures,equinus:ti,ab OR contracture,equinus:ti,ab OR 'equinus contractures':ti,ab OR 'talipes equinovalgus':ti,ab OR equinovalgus,talipes:ti,ab OR 'talipes valgus':ti,ab OR valgus,talipes:ti,ab OR splayfoot:ti,ab OR 'flat feet':ti,ab OR feet,flat:ti,ab OR 'flat foot':ti,ab OR 'pes planus':ti,ab OR foot,flat:ti,ab OR flatfeet:ti,ab OR 'vertical talus':ti,ab OR 'rigid flatfoot':ti,ab OR talus,vertical:ti,ab OR flatfoot,rigid:ti,ab OR 'foot,rocker bottom':ti,ab OR 'rocker bottom foot':ti,ab OR 'pes valgus,congenital convex':ti,ab OR 'vertical talus,congenital':ti,ab OR 'congenital vertical talus':ti,ab OR 'talus,congenital vertical':ti,ab OR 'convex pes valgus':ti,ab OR 'pes valgus,convex':ti,ab OR foot,convex:ti,ab OR 'convex foot':ti,ab OR 'talipes calcaneovalgus':ti,ab OR calcaneovalgus,talipes:ti,ab OR 'acquired adult flatfoot deformity':ti,ab OR 'flexible flatfoot':ti,ab OR flatfoot,flexible:ti,ab OR polydactylies:ti,ab OR polydactylia:ti,ab OR hyperdactyly:ti,ab OR 'congenital hip dislocations':ti,ab OR 'dislocations,congenital hip':ti,ab OR 'hip dislocations,congenital':ti,ab OR 'congenital hip dislocation':ti,ab OR 'congenital hip displacement':ti,ab OR 'displacements,congenital hip':ti,ab OR 'congenital hip displacements':ti,ab OR 'hip displacements,congenital':ti,ab OR 'congenital hip dysplasia':ti,ab OR 'congenital hip dysplasias':ti,ab OR 'hip dysplasias,congenital':ti,ab OR 'dysplasias,congenital hip':ti,ab OR 'dislocation,congenital hip':ti,ab OR 'hip,dislocation of,congenital':ti,ab OR 'displacement,congenital hip':ti,ab OR 'dysplasia,congenital hip':ti,ab OR 'hip displacement,congenital':ti,ab OR 'hip dysplasia,congenital':ti,ab OR 'congenital dysplasia of the hip':ti,ab OR 'dislocation of hip,congenital':ti,ab OR 'hip dysplasia,congenital,nonsyndromic':ti,ab OR skeleton:ti,ab OR bones:ti,ab OR osteopathic:ti,ab OR orthopaedic:ti,ab OR 'child osteology':ti,ab OR 'orthopedic disorders':ti,ab OR 'orthopedic disorder':ti,ab OR 'musculoskeletal symptom*':ti,ab OR 'musculoskeletal pain':ti,ab OR 'musculoskeletal complaint*':ti,ab OR 'musculoskeletal disease':ti,ab OR 'orthopedic injur*':ti,ab OR 'orthopaedic injur*':ti,ab OR deformity:ti,ab | #8 | 330725 |
| Total for Musculoskeletal Diseases | #7 OR #8 | #9 | 4280998 |
| Overall | #3 AND #6 AND #9 | #10 | 1835 |

| Concepts | CINAHL search strategy | Research | Results |
| --- | --- | --- | --- |
| Pediatric (controlled vocabulary) | (MM "Child+") OR (MM "Infant+") OR (MM "Adolescence+") OR (MM "Pediatrics+") | S1 | 52,220 |
| Pediatric (free text) | TI ( adolescen* OR baby OR babies* OR boy OR boys OR toddler OR preschool* OR child* OR girl* OR infan* OR kid OR kids OR neonat* OR newborn* OR paediatric* OR pediatric* ) OR AB ( adolescen* OR baby OR babies* OR boy OR boys OR toddler OR preschool* OR child* OR girl* OR infan* OR kid OR kids OR neonat* OR newborn* OR paediatric* OR pediatric* ) | S2 | 957,739 |
| Total for Pediatric | S1 OR S2 | S3 | 966,045 |
| Social media (controlled vocabulary) | (MM "Social Media+") OR (MM "Social Networks") | S4 | 18,024 |
| Social media (free text) | TI ( New media OR Social Networking OR Social Networks OR Network, Social OR Social Network OR Social media OR Digital media OR Instagram OR internet media OR internet medium OR media of the network OR network media OR network medium OR online media OR Facebook OR Google OR youtube OR WhatsApp OR Tumblr OR Twitter OR linkedin OR Instagram OR Pinterest OR Snapchat OR reddit OR qzone OR weibo OR WeChat OR youku OR tudou OR Renren OR badoo OR Baidu OR orkut OR foursquare OR vine OR Vkontakte OR myspace OR hi5 OR tiktok OR tik tok OR bebo) OR AB ( New media OR Social Networking OR Social Networks OR Network, Social OR Social Network OR Social media OR Digital media OR Instagram OR internet media OR internet medium OR media of the network OR network media OR network medium OR online media OR Facebook OR Google OR youtube OR WhatsApp OR Tumblr OR Twitter OR linkedin OR Instagram OR Pinterest OR Snapchat OR reddit OR qzone OR weibo OR WeChat OR youku OR tudou OR Renren OR badoo OR Baidu OR orkut OR foursquare OR vine OR Vkontakte OR myspace OR hi5 OR tiktok OR tik tok OR bebo) | S5 | 76,403 |
| Total for New media | S4 OR S5 | S6 | 80,697 |
| Musculoskeletal Diseases(controlled vocabulary) | (MM "Equinus Deformity") OR (MM "Flatfoot") OR (MM "Hip Dislocation, Congenital") OR (MM "Developmental Dysplasia of the Hip") OR (MM "Polydactyly+") OR (MM "Scoliosis+") OR (MM "Skeleton+") OR (MM "Bone and Bones+") OR (MM "Orthopedics") OR (MM "Musculoskeletal Diseases+") | S7 | 343,932 |
| Musculoskeletal Diseases(free text) | TI ( Equinus Deformity OR Genu Varum OR Flatfoot OR Polydactyly OR Scoliosis OR Hip Dislocation, Congenital OR Bow Leg OR Bow Legs OR Leg, Bow OR Legs, Bow OR Genu Varus OR Deformities, Equinus OR Deformity, Equinus OR Equinus Deformities OR Talipes Equinus OR Equinus Contracture OR Contractures, Equinus OR Contracture, Equinus OR Equinus Contractures OR Talipes Equinovalgus OR Equinovalgus, Talipes OR Talipes Valgus OR Valgus, Talipes OR Splayfoot OR Flat Feet OR Feet, Flat OR Flat Foot OR Pes Planus OR Foot, Flat OR Flatfeet OR Vertical Talus OR Rigid Flatfoot OR Talus, Vertical OR Rocker-Bottom Foot OR Flatfoot, Rigid OR Foot, Rocker-Bottom OR Rocker Bottom Foot OR Pes Valgus, Congenital Convex OR Vertical Talus, Congenital OR Congenital Vertical Talus OR Talus, Congenital Vertical OR Convex Pes Valgus OR Pes Valgus, Convex OR Foot, Convex OR Convex Foot OR Talipes Calcaneovalgus OR Calcaneovalgus, Talipes OR Acquired Adult Flatfoot Deformity OR Flexible Flatfoot OR Flatfoot, Flexible OR Polydactylies OR Polydactylia OR Hyperdactyly OR Congenital Hip Dislocations OR Dislocations, Congenital Hip OR Hip Dislocations, Congenital OR Congenital Hip Dislocation OR Congenital Hip Displacement OR Displacements, Congenital Hip OR Congenital Hip Displacements OR Hip Displacements, Congenital OR Congenital Hip Dysplasia OR Congenital Hip Dysplasias OR Hip Dysplasias, Congenital OR Dysplasias, Congenital Hip OR Dislocation, Congenital Hip OR Hip, Dislocation Of, Congenital OR Displacement, Congenital Hip OR Dysplasia, Congenital Hip OR Hip Displacement, Congenital OR Hip Dysplasia, Congenital OR Congenital Dysplasia Of The Hip OR Dislocation Of Hip, Congenital OR Hip Dysplasia, Congenital, Nonsyndromic OR skeleton OR bones OR osteopathic OR orthopaedic OR child osteology OR musculoskeletal disease OR orthopedic disorders OR orthopedic disorder OR musculoskeletal symptom* OR musculoskeletal pain OR musculoskeletal complaint* OR orthopedic injur* OR orthopaedic injur* OR deformity ) OR AB ( Equinus Deformity OR Genu Varum OR Flatfoot OR Polydactyly OR Scoliosis OR Hip Dislocation, Congenital OR Bow Leg OR Bow Legs OR Leg, Bow OR Legs, Bow OR Genu Varus OR Deformities, Equinus OR Deformity, Equinus OR Equinus Deformities OR Talipes Equinus OR Equinus Contracture OR Contractures, Equinus OR Contracture, Equinus OR Equinus Contractures OR Talipes Equinovalgus OR Equinovalgus, Talipes OR Talipes Valgus OR Valgus, Talipes OR Splayfoot OR Flat Feet OR Feet, Flat OR Flat Foot OR Pes Planus OR Foot, Flat OR Flatfeet OR Vertical Talus OR Rigid Flatfoot OR Talus, Vertical OR Rocker-Bottom Foot OR Flatfoot, Rigid OR Foot, Rocker-Bottom OR Rocker Bottom Foot OR Pes Valgus, Congenital Convex OR Vertical Talus, Congenital OR Congenital Vertical Talus OR Talus, Congenital Vertical OR Convex Pes Valgus OR Pes Valgus, Convex OR Foot, Convex OR Convex Foot OR Talipes Calcaneovalgus OR Calcaneovalgus, Talipes OR Acquired Adult Flatfoot Deformity OR Flexible Flatfoot OR Flatfoot, Flexible OR Polydactylies OR Polydactylia OR Hyperdactyly OR Congenital Hip Dislocations OR Dislocations, Congenital Hip OR Hip Dislocations, Congenital OR Congenital Hip Dislocation OR Congenital Hip Displacement OR Displacements, Congenital Hip OR Congenital Hip Displacements OR Hip Displacements, Congenital OR Congenital Hip Dysplasia OR Congenital Hip Dysplasias OR Hip Dysplasias, Congenital OR Dysplasias, Congenital Hip OR Dislocation, Congenital Hip OR Hip, Dislocation Of, Congenital OR Displacement, Congenital Hip OR Dysplasia, Congenital Hip OR Hip Displacement, Congenital OR Hip Dysplasia, Congenital OR Congenital Dysplasia Of The Hip OR Dislocation Of Hip, Congenital OR Hip Dysplasia, Congenital, Nonsyndromic OR skeleton OR bones OR osteopathic OR orthopaedic OR child osteology OR musculoskeletal disease OR orthopedic disorders OR orthopedic disorder OR musculoskeletal symptom* OR musculoskeletal pain OR musculoskeletal complaint* OR orthopedic injur* OR orthopaedic injur* OR deformity) | S8 | 209,546 |
| Total for Musculoskeletal Diseases | S7 OR S8 | S9 | 460,954 |
| Overall | S3 AND S6 AND S9 | S10 | 275 |

| Concepts | Cochrane Library search strategy | Research | Results |
| --- | --- | --- | --- |
| Pediatric | MeSH descriptor: [Child] explode all trees | #1 | 78477 |
|  | MeSH descriptor: [Infant] explode all trees | #2 | 41997 |
|  | MeSH descriptor: [Adolescent] explode all trees | #3 | 125806 |
|  | MeSH descriptor: [Pediatrics] explode all trees | #4 | 1179 |
|  | (adolescen* OR baby OR babies* OR boy OR boys OR toddler OR preschool* OR child* OR girl* OR infan* OR kid OR kids OR neonat* OR newborn* OR paediatric* OR pediatric*):ti,ab,kw | #5 | 352129 |
|  | #1 OR #2 OR #3 OR #4 OR #5 | #6 | 352142 |
| Social media | MeSH descriptor: [Social Networking] explode all trees | #7 | 195 |
|  | MeSH descriptor: [Social Media] explode all trees | #8 | 582 |
|  | (New media OR Social Networking OR Social Networks OR Network, Social OR Social Network OR Social media OR Digital media OR Instagram OR internet media OR internet medium OR media of the network OR network media OR network medium OR online media OR Facebook OR Google OR youtube OR WhatsApp OR Tumblr OR Twitter OR linkedin OR Instagram OR Pinterest OR Snapchat OR reddit OR qzone OR weibo OR WeChat OR youku OR tudou OR Renren OR badoo OR Baidu OR orkut OR foursquare OR vine OR Vkontakte OR myspace OR hi5 OR tiktok OR tik tok OR bebo):ti,ab,kw | #9 | 14388 |
|  | #7 OR #8 OR #9 | #10 | 14389 |
| Musculoskeletal Diseases | MeSH descriptor: [Genu Varum] explode all trees | #11 | 22 |
|  | MeSH descriptor: [Equinus Deformity] explode all trees | #12 | 53 |
|  | MeSH descriptor: [Flatfoot] explode all trees | #13 | 118 |
|  | MeSH descriptor: [Developmental Dysplasia of the Hip] explode all trees | #14 | 129 |
|  | MeSH descriptor: [Polydactyly] explode all trees | #15 | 3 |
|  | MeSH descriptor: [Scoliosis] explode all trees | #16 | 651 |
|  | MeSH descriptor: [Skeleton] explode all trees | #17 | 26481 |
|  | MeSH descriptor: [Bone and Bones] explode all trees | #18 | 17785 |
|  | MeSH descriptor: [Orthopedics] explode all trees | #19 | 556 |
|  | MeSH descriptor: [Musculoskeletal Diseases] explode all trees | #20 | 57508 |
|  | (Equinus Deformity OR Genu Varum OR Flatfoot OR Polydactyly OR Scoliosis OR Hip Dislocation, Congenital OR Bow Leg OR Bow Legs OR Leg, Bow OR Legs, Bow OR Genu Varus OR Deformities, Equinus OR Deformity, Equinus OR Equinus Deformities OR Talipes Equinus OR Equinus Contracture OR Contractures, Equinus OR Contracture, Equinus OR Equinus Contractures OR Talipes Equinovalgus OR Equinovalgus, Talipes OR Talipes Valgus OR Valgus, Talipes OR Splayfoot OR Flat Feet OR Feet, Flat OR Flat Foot OR Pes Planus OR Foot, Flat OR Flatfeet OR Vertical Talus OR Rigid Flatfoot OR Talus, Vertical OR Rocker-Bottom Foot OR Flatfoot, Rigid OR Foot, Rocker-Bottom OR Rocker Bottom Foot OR Pes Valgus, Congenital Convex OR Vertical Talus, Congenital OR Congenital Vertical Talus OR Talus, Congenital Vertical OR Convex Pes Valgus OR Pes Valgus, Convex OR Foot, Convex OR Convex Foot OR Talipes Calcaneovalgus OR Calcaneovalgus, Talipes OR Acquired Adult Flatfoot Deformity OR Flexible Flatfoot OR Flatfoot, Flexible OR Polydactylies OR Polydactylia OR Hyperdactyly OR Congenital Hip Dislocations OR Dislocations, Congenital Hip OR Hip Dislocations, Congenital OR Congenital Hip Dislocation OR Congenital Hip Displacement OR Displacements, Congenital Hip OR Congenital Hip Displacements OR Hip Displacements, Congenital OR Congenital Hip Dysplasia OR Congenital Hip Dysplasias OR Hip Dysplasias, Congenital OR Dysplasias, Congenital Hip OR Dislocation, Congenital Hip OR Hip, Dislocation Of, Congenital OR Displacement, Congenital Hip OR Dysplasia, Congenital Hip OR Hip Displacement, Congenital OR Hip Dysplasia, Congenital OR Congenital Dysplasia Of The Hip OR Dislocation Of Hip, Congenital OR Hip Dysplasia, Congenital, Nonsyndromic OR skeleton OR bones OR osteopathic OR orthopaedic OR child osteology OR musculoskeletal disease OR orthopedic disorders OR orthopedic disorder OR musculoskeletal symptom* OR musculoskeletal pain OR musculoskeletal complaint* OR orthopedic injur* OR orthopaedic injur* OR deformity):ti,ab,kw | #21 | 35565 |
|  | #11 OR #12 OR #13 OR #14 OR #15 OR #16 OR #17 OR #18 OR #19 OR #20 OR #21 | #22 | 97401 |
| Overall | #5 AND #10 AND #22 | #23 | 95 |
